# Ovarian fibroma torsion mimicking solid malignant tumors of the ovary: A case report and mini-review of the literature

**DOI:** 10.3892/mi.2025.280

**Published:** 2025-10-29

**Authors:** Efthymia Thanasa, Anna Thanasa, Emmanouil Xydias, Ioannis-Rafail Antoniou, Apostolos Ziogas, Ioannis Thanasas

**Affiliations:** 1 Department of Health Sciences, Medical School, Aristotle University of Thessaloniki, 54124 Thessaloniki, Greece; 2 Department of Obstetrics and Gynecology, EmbryoClinic IVF, 55133 Thessaloniki, Greece; 3 Department of Obstetrics and Gynecology, General Hospital of Trikala, 42100 Trikala, Greece; 4 Department of Medicine, University of Thessaly, School of Health Sciences, 41334 Larissa, Greece

**Keywords:** ovarian fibroma, torsion, solid ovarian cancer, preoperative diagnosis, differential diagnosis, frozen section, surgical management

## Abstract

The present study describes a rare case of torsion of an ovarian fibroma in a woman of reproductive age, where a severe diagnostic dilemma arose in differentiating it from a solid ovarian cancer. A 48-year-old female patient presented to the hospital with a 1-week history of low-grade fever and mild, continuous abdominal pain, accompanied by nausea and a mild sensation of abdominal bloating. The pain, which was not associated with signs of peritoneal irritation, was more pronounced in the right iliac fossa. The levels of inflammatory and tumor markers were found to be slightly elevated. A computed tomography scan and magnetic resonance imaging of the right adnexal region revealed a large heterogeneous mass with cystic-necrotic areas inside, free fluid collection in the pelvis, and multiple slightly enlarged para-aortic and iliac lymph nodes. These imaging findings, combined with the lack of clinical and laboratory improvement following treatment with antibiotics, led to the decision to proceed with a laparotomy, without having ruled out the diagnosis of solid ovarian cancer. Intraoperatively, the torsion of an ovarian fibroma was identified. A total abdominal hysterectomy with bilateral salpingo-oophorectomy was performed. An immunohistochemical examination confirmed the diagnosis of ovarian fibroma. Following an uneventful post-operative course, the patient was discharged from the clinic on the 4th day post-operatively. The present case report highlights an unusual clinical manifestation of ovarian fibroma torsion, which entailed milder symptom intensity and characteristics resembling ovarian malignancy instead. The aim of the present study was to report this uncommon manifestation, explore its differential diagnosis from malignancy and provide a brief review of the current diagnostic and treatment algorithms for clinicians faced with similar cases in their practice. In this manner, it is hoped that the present study may contribute to the improvement of the diagnostic accuracy and treatment efficacy for similar cases.

## Introduction

The ovaries are characterized by the occurrence of neoplasms with diverse clinical, morphological and histological features, and their pre-operative evaluation is considered to greatly contribute to the diagnostic approach and further management of these tumors (1). In the majority of cases (80%), ovarian tumors are benign and typically occur in young women between the ages of 20 and 45 years. The most common benign ovarian neoplasms are epithelial tumors, which are usually diagnosed in women of reproductive age. Malignant ovarian neoplasms, which account for ~20% of cases, are more commonly found in older women, between the ages of 40 and 65 years, and are generally associated with a poor prognosis (2). A distinct category of epithelial ovarian tumors are borderline malignant tumors, whose characteristics fall between those of benign and malignant categories (3).

Fibroma, thecoma and fibrothecoma are benign ovarian stromal tumors characterized by the presence of fibroblastic stromal cells and/or cells resembling luteinized theca cells (4). Ovarian fibromas, first described in 1983 by Young and Scully (5), are the most frequent solid ovarian neoplasms and account for ~1 to 4.7% of ovarian tumors (6). They are usually unilateral, non-hormone producing, and in most cases are diagnosed during the fifth to sixth decade of life in peri-menopausal or menopausal women (7). In some patients, ovarian fibromas, particularly when large, can lead to severe complications, such as torsion of the ipsilateral adnexa, necessitating immediate surgical intervention (6).

The present study describes a rare case of torsion of a large ovarian fibroma in a woman of reproductive age, which did not manifest with the expected intensity of torsion; it rather mimicked several clinical, biochemical and imaging features of a solid ovarian carcinoma. The aim of the present case report was to highlight this uncommon manifestation, explore its differential diagnosis from malignancy and provide a brief review of the current diagnostic and treatment algorithms for this condition, in order to assist clinicians encountering similar conditions in their practice.

## Case report

A 48-year-old woman with a history of four full-term vaginal deliveries, presented to the Emergency Department of the General Hospital of Trikala, Trikala, Greece, with a 1-week history of low-grade fever and abdominal pain. The pain was located in the lower abdomen, radiating mainly to the right iliac fossa. It was of mild intensity, continuous in nature, and accompanied by nausea and a mild sensation of abdominal distension. The pain was not associated with vomiting or diarrhea. The low-grade fever reportedly began ~2 days following the onset of the abdominal pain. The patient had an elevated body mass index (29.3) and a regular menstrual cycle. She had not visited a gynecologist in the past 18 years, following her last childbirth. Her medical history included hypothyroidism and arterial hypertension, both of which were well-controlled with medication. The patient also reported having undergone an appendectomy ~20 years prior.

**Figure 1:**
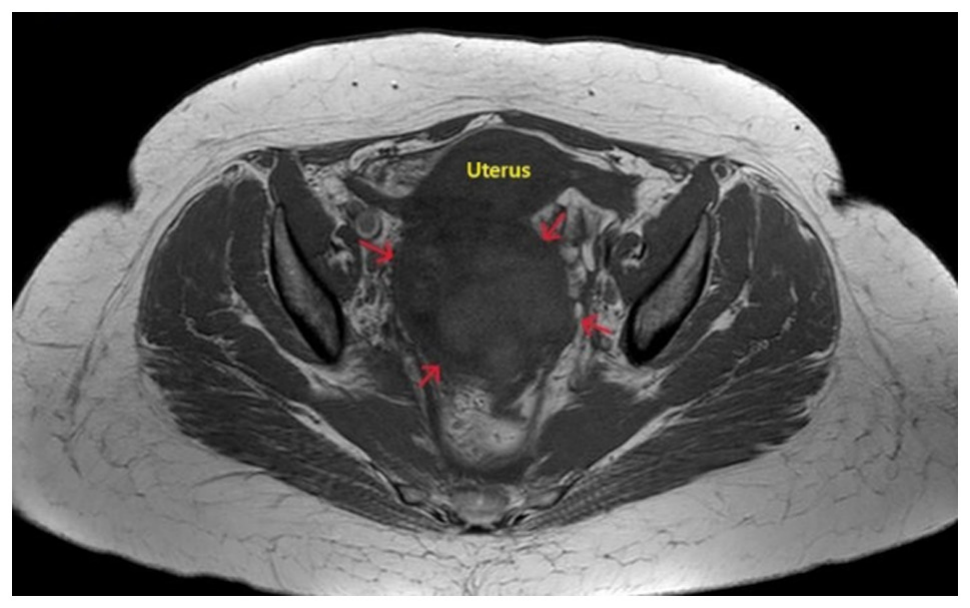
Magnetic resonance imaging of a twisted large ovarian fibroma: A large heterogeneous lesion (red arrows) is clearly visible in the region of the right adnexa, with cystic and necrotic areas present within.

Upon a clinical examination, her body temperature was found to be 37.7˚C, and her blood pressure and pulse rate were within normal range (125/75 mmHg and 85 beats/min, respectively). Palpation of the abdomen revealed mild tenderness, particularly in the right iliac fossa, without any signs of peritoneal irritation. The levels of inflammatory markers were found to be slightly elevated, as were the levels of tumor markers (Table I). The findings from the transvaginal and transabdominal ultrasound were inconclusive. A subsequent computed tomography (CT) scan revealed a large, well-circumscribed solid mass with thick walls, located between the rectosigmoid colon, the large bowel and the uterus, without contrast enhancement of its interior, but with wall and septal enhancement. Magnetic resonance imaging (MRI) of the right adnexal region demonstrated a large heterogeneous lesion measuring ~10x8.5 cm, with cystic and necrotic areas inside, as well as regions of intermediate-to-high signal intensity on T1 and low signal on T2 sequences (Fig. 1). The left ovary appeared normal. Additionally, there was diffuse fat stranding in the pelvic cavity, free fluid collection, and the presence of multiple mildly enlarged para-aortic and iliac lymph nodes. All investigations were inconclusive as regards the presence or absence of malignancy.

**Figure 2:**
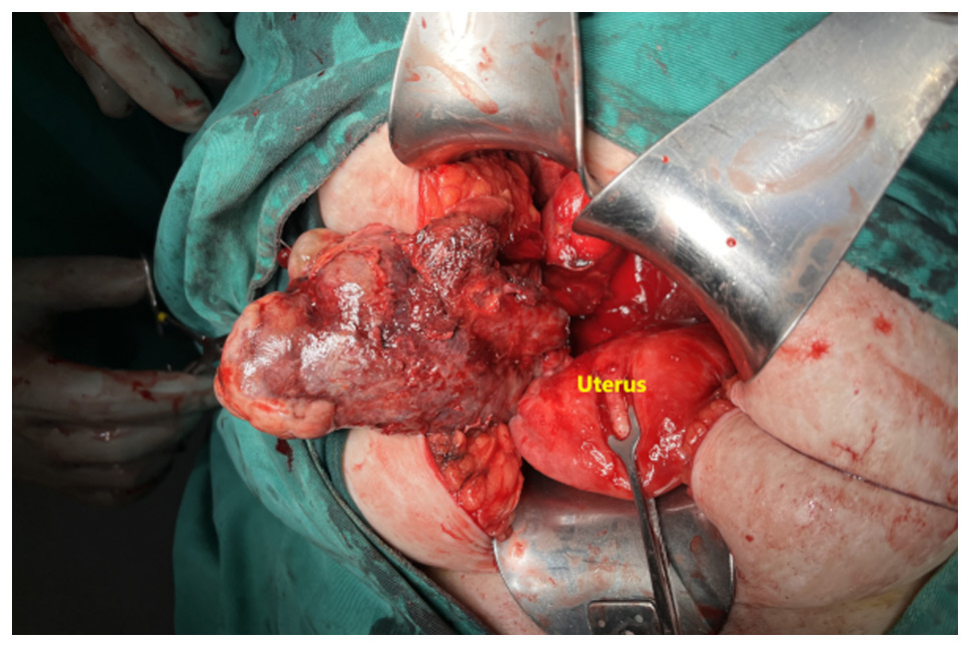
Intraoperative imaging of the ovarian fibroma with torsion.

**Figure 3:**
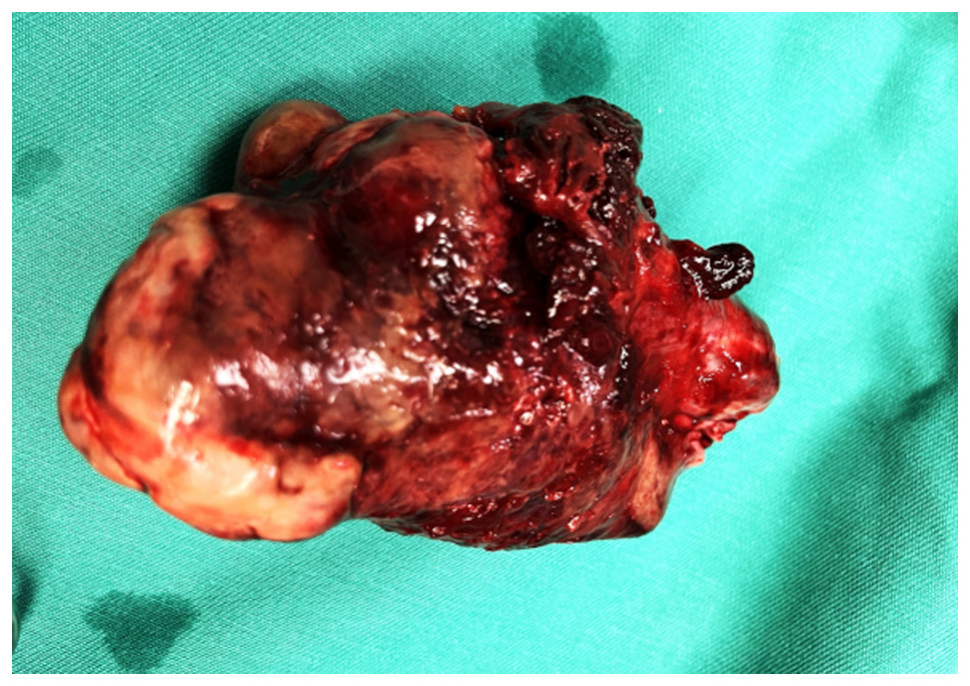
Surgical specimen of the twisted ovarian fibroma.

The combination of imaging findings, persistent clinical symptoms and the continued elevation of the levels of inflammatory markers despite intravenous treatment with antibiotics [cefoxitin (Mefoxil^®^) at a dose of 2 g every 8 h and metronidazole (Flagyl^®^) 500 mg three times a day], led to the decision to perform a laparotomy, without having excluded the diagnosis of solid ovarian cancer. Intraoperatively, a solid ovarian mass with torsion and marked inflammation was identified (Fig. 2). A frozen section indicated a benign tumor; however, this did not allow for definitive tumor classification. A total hysterectomy with bilateral salpingo-oophorectomy was performed. A macroscopic histological examination revealed a solid ovarian mass measuring 11x8x6.5 cm with a lobulated brown outer surface. Sectioning of the mass revealed a firm, elastic texture with areas of hemorrhage, findings consistent with an ovarian fibroma (Fig. 3).

**Figure 4:**
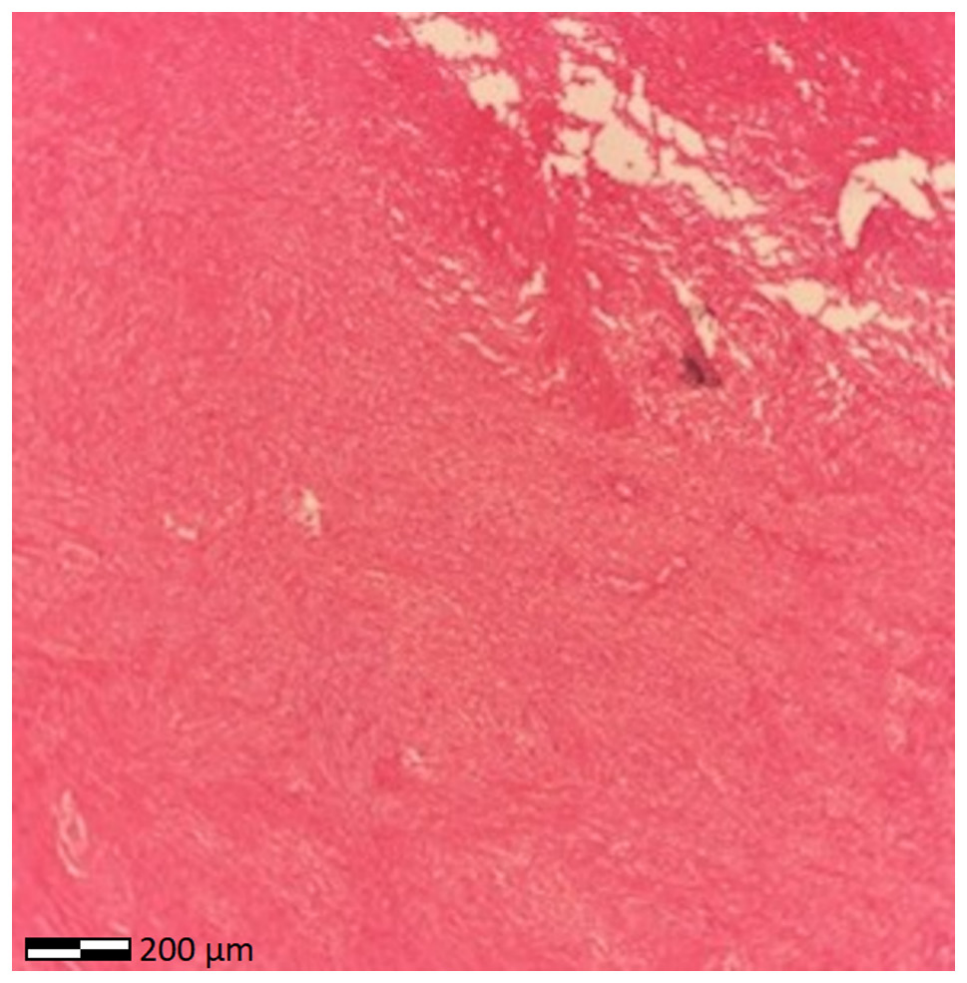
Histological image of ovarian fibroma torsion: Vascular congestion, hemorrhagic infiltration and ischemic necrosis of the ovarian tumor support the diagnosis of torsion of the corresponding adnexa (magnification, x100).

The specimens were subsequently sent for histopathological assessment at the laboratory of the hospital. As per the routine protocol, the specimens were embedded in paraffin cubes and 5-µm-thick sections were obtained for the analysis. A buffered, 10% formalin solution was utilized as a fixative medium, for 36 h at room temperature. Hematoxylin and eosin 0.5% alcohol (Diachel A.E.) staining was used, at room temperature with a 12-min duration. All microscopic examinations were performed using a LEICA DM2000 optical microscope (Leica Microsystems GmbH). The histological examination of the surgical specimen confirmed the diagnosis of pedunculated submucosal uterine leiomyoma (Fig. 3). A microscopic examination revealed vascular congestion, hemorrhagic infiltration and ischemic necrosis of the ovarian tumor, indicative of adnexal torsion (Fig. 4).

In addition, an immunohistochemical analysis was performed. The sections used were 4-µm-thick, were paraffin-embedded and were dewaxed for 40 min at 70˚C. The analysis was performed using the automated BOND-LEICA system (Leica Biosystems). The sections were placed sequentially in BOND Dewax solution, 100% v/v ethanol solution and BOND wash solution. For reticulin staining, the Reticulin Stain kit (cat. no. 25094, Polysciences) was used in accordance with the manufacturer's instructions. Oxidizer solution (1% potassium permanganate, Reticulin Stain kit, cat. no. 25094, Polysciences) was applied for 5 min at room temperature and subsequently the slices were rinsed in distilled water twice for 1 min at a time. The same procedure (solution bath and rinsing) was performed with a decolorizer/reducer solution (1% oxalic acid, Reticulin Stain kit, cat. no. 25094, Polysciences) and sensitizer solution (2.5% ferric ammonium sulfate, Reticulin Stain kit, cat. no. 25094, Polysciences). Finally, the slices were ready for silver impregnation, via a bath in a silver working solution (10% silver nitrate, 30% ammonium hydroxide and 3% sodium hydroxide, Reticulin Stain kit, cat. no. 25094, Polysciences) for 20 min and subsequent rinsing as described above. The slices were bathed in a developer solution (10% formalin) for 4 min, rinsed, bathed in a fixing solution (5% sodium thiosulphate) for 2 min at room temperature, rinsed and counterstained with (Reticulin Stain kit, cat. no. 25094, Polysciences) for 5 min at room temperature, before being rinsed, dehydrated in graded alcohols and mounted for microscopy (see below).

**Figure 5:**
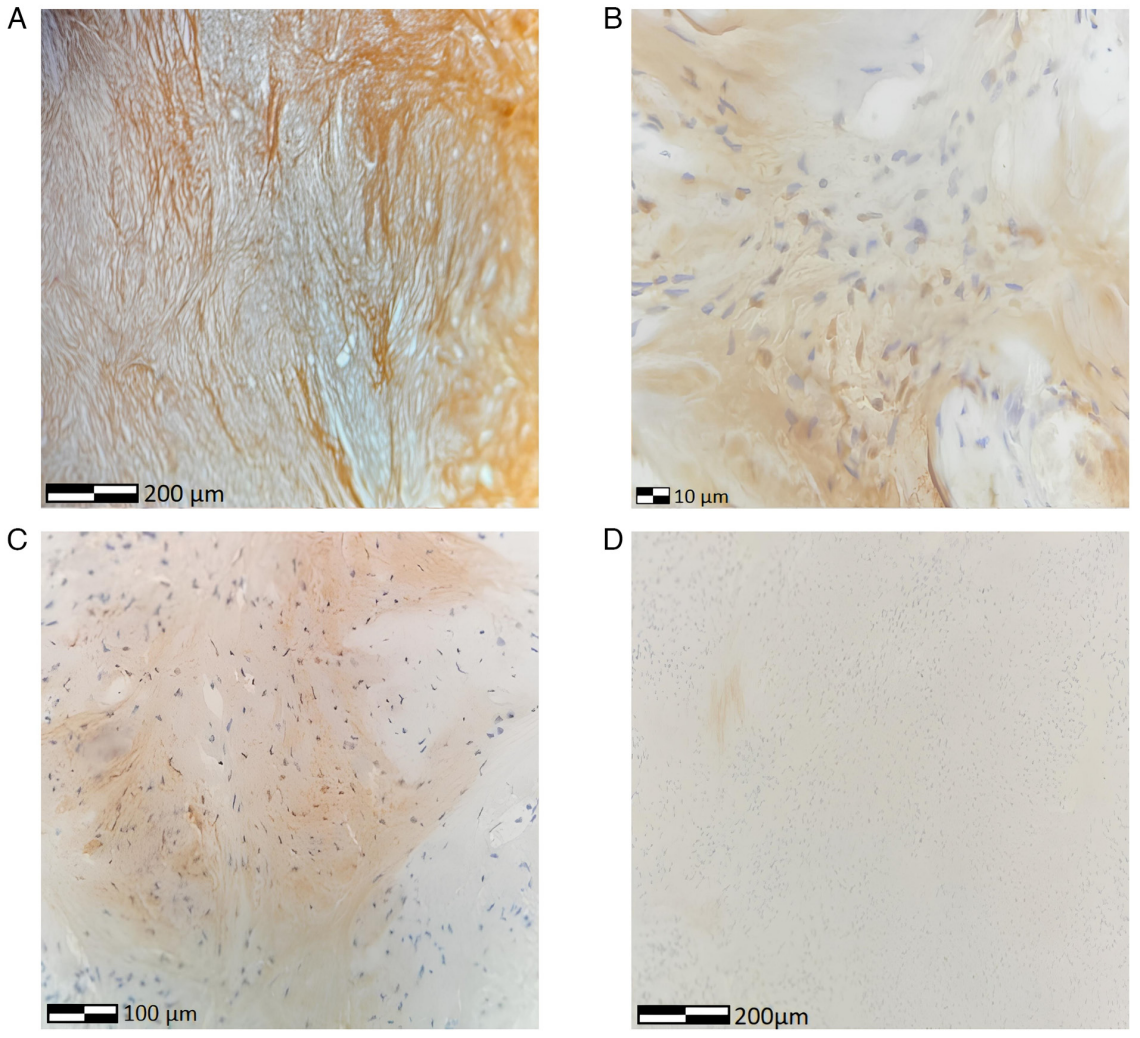
Immunohistochemical images of twisted ovarian fibroma: (A) Reticulin positivity (magnification, x100), (B) WT1 positivity (magnification, x400), (C) inhibin focal positivity (magnification, x400), (D) calretinin negativity (magnification, x40).

For antigen retrieval, BOND proprietary Heat Induced Epitope Retrieval (HIER) solution was used for 20 min in 100˚C and pH 8. The block peroxide kit (Bond; Leica Biosystems) was subsequently used for 5 min. For WT1, the anti-WT1 antibody (cat. no. AMAb91842, Atlas Antibodies) was used at a 1:1,200 dilution. For inhibin, the 27331-1-AP antibody (cat. no. Ag25999, Proteintech Group, Inc.) was used at a 1:800 dilution. For calretinin, the 82811-1-RR antibody (cat. no. Ag2924, Proteintech Group, Inc.) was used at a 1:400 dilution. For all antibodies, a duration of incubation of 30-60 min was used at 37^˚^C. Subsequently, a secondary detection kit polymer (Bond; Leica Biosystems) was used for a duration of 10 min with a post-primary (rabbit anti-mouse) step followed by the polymer anti-rabbit antibody (DS9800, Bond; Leica Biosystems), HRP-conjugated at room temperature for 8 min for WT1 antibody and polymer anti-rabbit antibody (DS9800, Bond; Leica Biosystems), HRP-conjugated at room temperature for 8 min for inhibin and calretinin. All secondary antibodies were ready-to-use and required no dilution. The DAB kit (Bond; Leica Biosystems) was also used for 10 min to facilitate visualization. Hematoxylin was applied for 5 min as a counterstain at room temperature and the sections were dehydrated, mounted and cover-slipped. The resulting slides were examined under a LEICA DM2000 optical microscope (Leica Microsystems GmbH), at a magnification of x40, x100 and x400. Immunohistochemical analysis confirmed the diagnosis of ovarian fibroma (Fig. 5).

The post-operative course of the patient was uneventful (Table I), and the patient was discharged on the 4th day post-operatively day. At 1 month following surgery, the tumor marker levels were within normal limits.

## Discussion

The present study describes a case of ovarian fibroma torsion with manifestations resembling ovarian malignancy. The clinical diagnosis of twisted ovarian fibromas is challenging, as they can mimic those of malignant ovarian tumors or degenerating uterine leiomyomas (8). The sudden onset of acute abdominal pain, accompanied by discomfort, severe nausea and multiple episodes of vomiting, constitutes the primary clinical feature commonly observed in patients with torsion of a large ovarian fibroma (9). In the patient described herein, the symptoms were considerably milder, with low-intensity abdominal pain for 12 days, not associated with signs of peritoneal irritation. The patient did not report any vomiting; instead, the main accompanying symptom of the abdominal pain was low-grade fever. Based on these clinical findings, the further evaluation of the patient with advanced imaging was warranted, rather than proceeding directly to emergency surgery, which is typically indicated in cases of clear indications of adnexal torsion and necrosis. Torsion, ischemia, and necrosis of the adnexa represent an emergency condition requiring immediate intervention due to the high risk of reactive peritonitis and ischemic gangrene (10). The lack of acute, worsening symptoms in the patient in the present study, necessitated the inclusion of a malignant ovarian mass in the differential diagnosis.

Beyond the unconventional clinical manifestations, the imaging findings were also far from definitive in the case in the present study. Ultrasonographic findings of ovarian fibromas are generally non-specific, as was the case in the patient described herein. It is estimated that transvaginal ultrasonography has a sensitivity, specificity and accuracy of 97, 46 and 68%, respectively, for the detection of solid adnexal masses (11). Doppler ultrasound imaging of the pelvis is currently considered particularly useful in the evaluation of adnexal torsion, being able to distinguish areas of reduced perfusion; however, such findings may be caused by both ischemia due to torsion and areas of necrosis within the tumor, as was the case in the patient in the present study (12). A CT scan cannot reliably differentiate ovarian fibromas from other solid ovarian masses, given its lacking visualization capabilities as regards soft tissues (12). The midline location of the ovarian mass, deviation of the uterus toward the affected ovary, and the presence of varying degrees of ascitic fluid are imaging findings that support the diagnosis of adnexal torsion (12). MRI is considered a second-line diagnostic tool, with substantial imaging capabilities for soft tissue lesions. Despite this fact, it is important to note that the pre-operative differentiation of ovarian fibromas from subserosal pedunculated uterine leiomyomas or solid malignant ovarian tumors remains difficult, even if an MRI is used (8). In the patient described herein, neither ultrasonography, nor CT, nor MRI could pre-operatively establish a diagnosis of a twisted ovarian fibroma or definitively differentiate it from solid ovarian cancer.

The differential diagnosis of ovarian fibroma, with or without torsion, from solid ovarian cancer is challenging, if based solely on clinical symptoms and conventional imaging, as established in the present case report. Recent data however, suggest that convolutional neural networks based on MRI may greatly aid in the non-invasive preoperative differentiation of these tumors (13). This advanced technique is capable of accurately identifying the nature of the mass (ovarian fibroma vs. solid ovarian carcinoma), thereby guiding appropriate treatment strategies (13). This utility would be even more helpful in rare cases of unilateral ovarian fibroma with ascites and pleural effusion, or in bilateral presentations with ascitic fluid, where differentiating it from Demons-Meigs syndrome or metastatic ovarian tumors pre-operatively would be of even greater clinical significance.

An accurate diagnosis is crucial to avoid mismanagement, which can result in increased morbidity and mortality rates (14-16). In cases such as the one described in the present study, in the absence of clear pre-operative diagnosis, a histopathological analysis is the only method that can be used to reach a definitive conclusion, using intra-operative frozen sections. Frozen sections can assist in confirming or eliminating the presence of malignancy and can thus guide intra- and post-operative treatment accordingly, as was the case in the present study. However, frozen sections should not constitute the only diagnostic investigation or be entirely relied upon for surgical decision-making, as they are associated with certain limitations (17). In particular, sampling errors, including poor biopsy site selection and poor assessment of cell infiltration; technical errors, including freezing artifacts and poor staining; and interpretive errors, due to tumor heterogeneity and uncertainty, indicate that frozen sections should not be considered infallible and render proper communication and coordination between surgeon and pathologist of vital importance (17). This is even more pronounced in cases where metastatic ovarian tumors originating from primary malignancies, typically of the gastrointestinal tract or breast, coexist with benign ovarian tumors such as fibromas, thecomas and fibrothecomas, posing severe diagnostic challenges, even with the utilization of frozen sections (18). Olaofe *et al* (19) reported a unique case of a Krukenberg tumor arising within an ovarian fibroma, where diagnosis was particularly difficult due to histological similarities between Krukenberg cells and ovarian stromal cells, particularly when present in limited foci. In the case described herein, although only a single tumor component was present, the frozen section was only able to exclude malignancy and could not provide any information regarding histological type or classification of the neoplasm, once more confirming the limitations of solely relying on this technique.

Finally, the utility of tumor markers in the diagnosis of ovarian fibromas should be discussed. Although rare, ovarian fibromas associated with elevated serum CA-125 levels should be included in the differential diagnosis with epithelial ovarian carcinoma, not only in post-menopausal women, but also in women of reproductive age (20). In the patient in the present study, the borderline elevated CA-125 levels led to the suspicion of malignancy; however, combined with the mild abdominal pain, persistent low-grade fever and elevated inflammatory markers, they may also be attributed to inflammatory conditions with ischemia and necrosis. Similarly, the borderline enlargement of multiple para-aortic and iliac lymph nodes, and the reported presence of reactive pelvic fluid collections may be attributed to either an inflammatory or malignant etiology, further contributing to the diagnostic dilemma between a twisted ovarian fibroma and solid ovarian carcinoma. Ultimately, definitive diagnosis was made via a scheduled surgical intervention.

The management of a twisted ovarian fibroma is surgical, either via laparotomy or laparoscopy. A laparoscopic approach is reserved for young patients with exophytic ovarian fibroma and minimal risk of intraperitoneal tumor dissemination (21). In the event that the damage to the twisted adnexa is reversible and the patient is of reproductive age with a desire for future fertility, the excision of the fibroma or, if necessary, unilateral salpingo-oophorectomy following intraoperative frozen section analysis is considered the treatment of choice (20,21). A total hysterectomy with bilateral salpingo-oophorectomy is indicated in older patients (22). In the patient in the present study, a total abdominal hysterectomy with bilateral salpingo-oophorectomy was performed. The patient expressed a lack of desire for future childbearing; thus, the spread of inflammation from the twisted adnexa to the uterus, and particularly the inconclusive intraoperative histological assessment of a presumably benign tumor requiring multiple permanent sections for definitive diagnosis, were the reasons for opting for total hysterectomy rather than adnexectomy.

In conclusion, ovarian fibromas are the most common solid ovarian tumors. Torsion of a large ovarian fibroma is a rare, yet severe complication that typically requires prompt surgical intervention. In the patient in the present study, the mild and non-alarming progression of the disease deviated from expected clinical manifestations and necessitated a complete preoperative work-up and the performance of a scheduled total abdominal hysterectomy with bilateral salpingectomy and oophorectomy. The solid nature of ovarian fibromas, their atypical clinical and imaging features, and their potential association with ascites and elevated serum CA-125 levels necessitate thorough differential diagnosis in order to eliminate the possibility of solid ovarian malignancy. Although the analysis of intraoperative frozen sections may suggest a likely benign tumor, definitive classification of the ovarian mass requires multiple permanent histological sections. Only following the surgical removal of the tumor and performing an immunohistochemical analysis can a definitive diagnosis of ovarian fibroma be established.

## Figures and Tables

**Table I:** Laboratory tests of the patient upon arrival at the emergency department and during hospitalization.

Laboratory tests	Day of arrival at the ED	1st Day of hospitalization	3rd Day of hospitalization	5th Day of hospitalization	1st Post-operative day	2nd Post-operative day	4th Post-operative day	1st Post-operative month	Normal laboratory values
Ht	37.4%	35.6%	34.3%	33.9%	30.1%	29.2%	30.7%	-	37.7-49.7%
Hb	12.9 g/dl	11.9 g/dl	11.1 g/dl	10.9 g/dl	9.9 g/dl	9.3 g/dl	10.1 g/dl	-	11.8-17.8 gr/dl
PLT	239x10^3/^ml	224x10^3/^ml	213 x10^3/^ml	210x10^3/^ml	201x10^3/^ml	205x10^3/^ml	217x10^3/^ml	-	150-350x10^3/^ml
WBC	17.8x10^3/^ml	15.3x10^3/^ml	14.1 x10^3/^ml	13.7x10^3/^ml	22.4x10^3/^ml	14.1x10^3/^ml	9.4x10^3/^ml	-	4-10.8x10^3/^ml
NEUT	84.4%	84.1%	83.2%	81.9%	92.9%	84.1%	73.2%	-	40-75%
CRP	25.70 mg/dl	26.99 mg/dl	22.55 mg/dl	19.95 mg/dl	19.91 mg/dl	10.32 mg/dl	4.37 mg/dl	-	<0.7 mg/dl
APTT	30.4 sec	30.9 sec	31.8 sec	33.9 sec	32.8 sec	30.7 sec	29.8 sec	-	24.0-35.0 sec
INR	1.25	1.27	1.28	1.37	1.41	1.25	1.17	-	0.8-1.2
FIB	421 mg/dl	437 mg/dl	447 mg/dl	565 mg/dl	513 mg/dl	491 mg/dl	373 mg/dl	-	200-400 mg/dl
Glu	112 mg/dl	85 mg/dl	91 mg/dl	85 mg/dl	91 mg/dl	85 mg/dl	84 mg/dl	-	75-115 mg/dl
Cr	0.62 mg/dl	0.56 mg/dl	0.63 mg/dl	0.66 mg/dl	0.67 mg/dl	0.63 mg/dl	0.51 mg/dl	-	0.40-1.10 mg/dl
Κ^+^	4.25 mmol/l	4.66 mmol/l	4.61 mmol/l	4.32 mg/dl	4.21 mg/dl	4.11 mg/dl	4.09 mg/dl	-	3.5-5.1 mmol/l
Να^+^	136.5 mmol/l	139.2 mmol/l	138.1 mmol/l	139.1 mmol/l	138.9 mmol/l	141.2 mmol/l	141.4 mmol/l	-	136-145 mmol/l
TBIL	0.60 mg/dl	0.48 mg/dl	-	-	0.51 mg/dl	-	-	-	0.3-1.2 mg/dl
DBIL	0.07 mg/dl	0.12 mg/dl	-	-	0.11 mg/dl	-	-	-	0.0-0.5 mg/dl
INBIL	0.27 mg/dl	0.31 mg/dl	-	-	0.35 mg/dl	-	-	-	0.0-0.7 mg/dl
SGOT	19 IU/l	17 IU/l	-	-	18 IU/l	-	-	-	5-33 IU/l
SGPT	18 IU/l	15 IU/l	-	-	19 IU/l	-	-	-	10-37 IU/l
AMY	21 IU/l	22 IU/l	-	-	21 IU/l	-	-	-	30-118 IU/l
CEA	5.1 ng/ml	-	-	-	-	-	-	0.8 ng/ml	<5 ng/ml
CA125	82.6 U/ml	-	-	-	-	-	-	7 U/ml	≤35 U/ml
CA15-3	31.4 U/ml	-	-	-	-	-	-	12.4 U/ml	0.0-31.3 U/ml
CA19-9	40.7 U/ml	-	-	-	-	-	-	3.3 U/ml	0.0-37 U/ml

Ht, hematocrit; Hb, hemoglobin; PLT, platelets; WBC, white blood cells; NEUT, neutrophils; CRP, C-reactive protein; APTT, activated partial thromboplastin time, INR, international normalized ratio; FIB, fibrinogen; Glu, glucose; Cr, creatinine; K^+^, potassium; Na^+^, sodium; TBIL, total bilirubin; DBIL, direct bilirubin; IDBIL, indirect bilirubin; SGOT, serum glutamic oxaloacetic transaminase; SGPT, serum glutamate pyruvate transaminase; AMY, amylase; CEA, carcinoembryonic antigen; CA125, cancer antigen 125; CA15-3, cancer antigen 15-3; CA19-9, cancer antigen 19-9.

## Data Availability

The data generated in the present study may be requested from the corresponding author.
